# Newly Designed PCL-Wrapped Cryogel-Based Conduit Activated with IKVAV Peptide Derivative for Peripheral Nerve Repair

**DOI:** 10.3390/pharmaceutics16121569

**Published:** 2024-12-08

**Authors:** Abdulla Yergeshov, Mohamed Zoughaib, Kenana Dayob, Marat Kamalov, Duong Luong, Albina Zakirova, Ruslan Mullin, Diana Salakhieva, Timur I. Abdullin

**Affiliations:** 1 Scientific and Educational Center of Pharmaceutics, Kazan (Volga Region) Federal University, 18 Kremlyovskaya St., 420008 Kazan, Russiazmokhamed@kpfu.ru (M.Z.); kdaiob@stud.kpfu.ru (K.D.);; 2 Institute of Fundamental Medicine and Biology, Kazan (Volga Region) Federal University, 18 Kremlyovskaya St., 420008 Kazan, Russia; 3 Institute for Molecular Bioscience, The University of Queensland, St. Lucia, QLD 4072, Australia; 4 Academy of Postgraduate Education under FSBU FSCC of FMBA of Russia, Department of Oncology and Plastic Surgery, 91 Volokolamsk Highway, 125371 Moscow, Russia; 5 State Autonomous Healthcare Institution Republican Clinical Hospital of the Ministry of Health of the Republic of Tatarstan, 138 Orenburg Highway, 420064 Kazan, Russia

**Keywords:** peripheral nerve repair, nerve conduits, cryogels, ECM proteins, synthetic peptides, IKVAV derivatives, polycaprolactone, neurogenic cells, sciatic nerve diastasis, tissue regeneration

## Abstract

**Background:** The combination of macroporous cryogels with synthetic peptide factors represents a promising but poorly explored strategy for the development of extracellular matrix (ECM)-mimicking scaffolds for peripheral nerve (PN) repair. **Methods:** In this study, IKVAV peptide was functionalized with terminal lysine residues to allow its in situ cross-linking with gelatin macromer, resulting in the formation of IKVAV-containing proteinaceous cryogels. The controllable inclusion and distribution of the peptide molecules within the scaffold was verified using a fluorescently labelled peptide counterpart. The optimized cryogel scaffold was combined with polycaprolactone (PCL)-based shell tube to form a suturable nerve conduit (NC) to be implanted into sciatic nerve diastasis in rats. **Results:** The NC constituents did not impair the viability of primary skin fibroblasts. Concentration-dependent effects of the peptide component on interrelated viscoelastic and swelling properties of the cryogels as well as on proliferation and morphological differentiation of neurogenic PC-12 cells were established, also indicating the existence of an optimal-density range of the introduced peptide. The in vivo implanted NC sustained the connection of the nerve stumps with partial degradation of the PCL tube over eight weeks, whereas the core-filling cryogel profoundly improved local electromyographic recovery and morphological repair of the nerve tissues, confirming the regenerative activity of the developed scaffold. **Conclusions:** These results provide proof-of-concept for the development of a newly designed PN conduit prototype based on IKVAV-activated cryogel, and they can be exploited to create other ECM-mimicking scaffolds.

## 1. Introduction

Peripheral nerve (PN) injury generally caused by mechanical trauma and accompanied by sensory-motor dysfunctions and chronic pain is a global health issue with profound impact on the quality of life of affected individuals and healthcare cost [1]. Transplantation of autologous nerve grafts remains the gold-standard treatment for long-gap PN lesions [2]. The autografts, along with their limited availability, have intrinsic drawbacks related to excision of the donor nerve, potential size mismatch, and variability of regenerative properties of the transplant. Similar limitations are shared by allograft counterparts, which additionally pose risks of immunogenic response and infectious disease transmission [3]. Although the latter problems can be partially overcome by using pre-decellularized grafts, these cadaver-derived biomaterials are poorly compliant with large-scale production in a reproducible manner and may have limited efficacy upon large PN defects [4].

Substitution of the nerve grafts with engineered nerve conduits (NCs) to bridge the PN gap and promote tissue repair is a worldwide challenge. Typically composed of biopolymers, synthetic polymers, and composites, NCs have evolved from biocompatible surgical wraps that draw together nerve stumps to extracellular matrix (ECM)-like scaffolds that support neural cells and guide axonal growth [5]. NCs can overcome the limitations of nerve grafts and provide better control over neuroma and scar formation [6]. However, current NC-forming biomaterials are inferior to natural ECMs and possess limited regenerative potential [7].

Among the different types of polymer scaffolds for neural regeneration, hydrogel materials with a hydrophilic cytocompatible nature and tunable mechanical properties seem preferable to develop ECM-mimicking NCs [8,9]. The state of the art in hydrogel materials for PN regeneration is summarized in recent reviews [10,11,12]. The prevalent constituent of these biomaterials is natural polymers, which are intrinsically biocompatible, biodegradable, and preserve biochemical similarity to intact ECM in different extents (see Discussion Section for some particular designs). There are some NC products particularly containing type I collagen, such as NeuraGen, Neuroflex, NeuroMatrix, NeuraWrap, and NeuroMend, which have been approved by the FDA [10,13,14,15,16].

A subclass of hydrogels termed cryogels can be advantageous compared to conventional hydrogel materials for tissue engineering owing to improved mechanical properties and a porous structure that better support motion, nutrient exchange, and other vital functions of the cells [17,18]. During recent years, the regenerative potential of cryogel-based NCs for PN repair has been revealed in vitro and in vivo [18,19,20,21,22].

To induce specific cellular responses and potentiate the repair process, neuroactive agents such as growth factors (GFs) or ECM proteins can be added to the composition of NC. The use of biospecific peptide motifs derived from these polypeptides/proteins offers several advantages over native sequences in terms of stability, purity, and cost-effectiveness. For instance, RGD, YIGSR, IKVAV ECM-derived peptides, and RGI BDNF-derived peptide, among others, have been identified and tested as active components of NCs [23,24,25].

Being one of the most relevant cell-specific motifs of laminin, IKVAV peptide plays an important role in cell–ECM interactions, signaling, and neurite outgrowth [26]. Increased cell viability and neurogenic differentiation were reported for neural stem cells (NSCs) cultured in IKVAV-modified silk fibroin [27] and RADA_16_-IKVAV self-assembling hydrogels [28]. Likewise, neural progenitor cells grown in amphiphilic IKVAV peptide nanofiber hydrogels differentiated into neuron-like and glial-like cells [29]. The combination of short ECM-derived peptides with cryogel scaffolds to treat PN injuries has not been reported yet.

Successful design of peptide-activated biomaterials requires an effective immobilization strategy. We previously proposed in situ affinity functionalization of cryogels with synthetic peptides to mimic the ECM environment [30,31]. Although chemical cross-linking provides one of the most robust ways to incorporate proteinaceous components into biomaterials, conventional short peptide sequences are not compliant with existing cross-linking methods and may undergo inactivation.

In this study, the IKVAV motif was rationally modified with additional lysine residues to both of its ends to allow its incorporation in a controllable manner into the model protein (gelatin) network during cryogel formation. The effects of the peptide component on the physical and biochemical characteristics of the functionalized material including neuronal cell responses were verified. The newly designed IKVAV–gelatin cryogel was combined with a firm polycaprolactone (PCL) shell tube to develop a prototype of implantable and suturable NC, which was tested in a rat sciatic nerve gap model. In addition to the proven neuroactivity of the designed composite NC, the reported methodology can be extended to comprise other ECM peptides and protein matrices for future tissue engineering applications.

## 2. Materials and Methods

### 2.1. Materials

Rink amide resin, Fmoc-Lys(Mtt)-OH, Fmoc-Ile-OH, Fmoc-Val-OH, Fmoc-Ala-OH, 1-pyrenebutyric acid, 2-(1H-benzotriazol-1-yl)-1,1,3,3-tetramethyluronium hexafluorophosphate (HBTU), N,N-diisopropylethylamine (DIPEA), triisopropylsilane (TIPS), trifluoroacetic acid (TFA), N,N-dimethylformamide (DMF), and dichloromethane (DCM) were purchased from Merck, Novabiochem, and TCI.

Cell culture media and reagents were purchased from PanEco (Moscow, Russia). A PC-12 rat pheochromocytoma cell line was obtained from the American Type Culture Collection. 3-(4,5-Dimethylthiazol-2-yl)-5-(3-carboxymethoxyphenyl)-2-(4-sulfophenyl)-2H-tetrazo-lium (MTS reagent) was purchased from Promega (Madison, WI, USA). Bovine skin gelatin, poly-ε-caprolactone (PCL, M_n_ ≈ 80 kDa), phenazine methosulfate (PMS), gold (III) chloride, silver nitrate, and bisBenzimidine trihydrochloride (Hoechst) H33342 were purchased from Sigma-Aldrich (St. Louis, MO, USA). Lecithin from egg yolk, glutaric aldehyde, p-formaldehyde, violet acetate, and luxol fast blue were purchased from Acros Organics (Antwerpen, Belgium). CellTracker Red CMTPX was supplied by ThermoFisher Scientific (Waltham, MA, USA). Giemsa and hematoxylin-eosin stains were purchased from BioVitrum (Saint-Petersburg, Russia). Tiletamine–zolazepam (Virbac, Carros, France), xylazine (Nita-Pharm, Saratov, Russia), and ketorolac (Public company ‘Sintez’, Kurgan, Russia) were used. Suture materials were obtained from ‘Medtekhnika’ Ltd. (Kazan, Russia).

### 2.2. Peptide Synthesis

KIKVAVK-NH_2_ peptide with a C-terminal amide group was synthesized by Fmoc solid-phase peptide synthesis (SPPS) method as detailed previously [32]. Briefly, following swelling of Fmoc-Rink amide resin in DMF and deprotection in 20% (*v*/*v*) piperidine (in DMF), subsequent coupling and deprotection cycles were carried out using 2 equiv. of Fmoc-protected amino acids, 1.98 equiv. of 2-(1H-benzotriazol-1-yl)-1,1,3,3-tetramethyluronium hexafluorophosphate (HBTU), and 3 equiv. of N,N-diisopropylethylamine (DIPEA) in DMF. The peptides were cleaved from the resin using 95% trifluoroacetic acid (TFA), 2.5% H_2_O, and 2.5% triisopropylsilane (TIPS) mixture, and collected with dichloromethane (DCM), which was evaporated alongside TFA on a rotary evaporator. Cold diethyl ether was used for trituration, then the peptides were sedimented by centrifugation, dissolved in deionized water, and lyophilized. For fluorescence labeling of the peptide, pyrenebutyric acid was introduced via ε-amino group of Lys residue after removal of its protecting Mtt (4-methyltrityl) group with 5% TFA cleavage cocktail in DCM.

The structure and purity of the synthesized peptides were confirmed by reverse-phase HPLC and mass-spectrometric (MS) analysis. A Dionex UltiMate 3000 chromatograph (Thermo Scientific, Waltham, MA, USA) and a ZORBAX SB-C18 column (5 μm, 4.6 × 150 mm) (Agilent Technologies, Santa Clara, CA, USA) were used. The mobile phase contained (A) acetonitrile with 0.1% trifluoroacetic acid, and (B) milli-Q water with 0.1% trifluoroacetic acid. The gradient scheme was as follows: 0–30 min: from 2% A to 2% B, 30–40 min: from 98% A to 98% B. The flow rate was 0.5 mL/min, and the injection volume was 10 μL. MS measurements were performed on a TripleTOF 5600 (AB SCIEX) triple quadrupole-time-of-flight mass spectrometer with electrospray ionization. The data are shown in Figure S1.

### 2.3. Preparation and Characterization of Polycaprolactone-Based Film

PCL and lecithin were co-dissolved in dichloromethane at a mass ratio of 3.6:1. The mixed solution was poured into a Petri dish, and the solvent was allowed to evaporate at ambient atmosphere, resulting in the formation of a thin polymer film. The film was additionally kept in a rotary vacuum evaporator to remove residual amounts of the solvent. The resulting material was cut into rectangular pieces, which were rolled into tubes and sealed by etching the contacting edges of the film.

The surface of the materials was analyzed using scanning electron microscopy (SEM). The samples were coated with a 15 nm conductive layer (Au-Pd alloy) by cathodic sputtering on a Q150T ES coater (Quorum Technologies, Lewes, UK). SEM analysis was performed on a Merlin (Carl Zeiss, Oberkochen, Germany) high-resolution scanning electron microscope at an acceleration voltage of 5 kV and a probe current of 300 pA. The maximum pull force upon tensile failure of the tubed materials was analyzed using an AMITTARI FG-104-1K digital force gauge.

### 2.4. Fabrication and Characterization of Composite Conduit

#### 2.4.1. Preparation of Cryogel and Conduit

The cryogel materials were prepared by cross-linking of gelatin in an aqueous solution with glutaric aldehyde under subzero temperature. Furthermore, a series of composite cryogels were obtained by copolymerization of the gelatin (2.5 wt.%) with KIKVAVK peptide or its fluorescent derivative (0.001–0.125 wt.%) in a mixed solution. The formed cryogels were kept in a concentrated penicillin/streptomycin solution for decontamination then washed in sterile water several times prior to use. The relative peptide content in the resultant cryogels (0.04, 0.1, 0.5 wt.%, per dry matrix) was estimated by assuming the total entrapment of both gelatin and peptide in the materials. For in vitro study, the cryogels were formed as 2 mm thick flat membranes. To produce the conduit, the pre-optimized cryogel material was formed under the same conditions but inside a PCL–lecithin tube.

#### 2.4.2. FTIR and DSC Analyses

The attenuated total reflectance-Fourier transform infrared (ATR–FTIR) spectra of PCL-based films were recorded on a Frontier spectrometer (PerkinElmer, Waltham, MA, USA) in the wavenumber range 4000–500 cm^–1^ with a resolution of 1 cm^–1^. Differential scanning calorimetry (DSC) was carried out using an STA 449 Jupiter thermal analyzer (Netzsch, Selb, Germany). The conditions for the latter analysis were as detailed previously [33].

#### 2.4.3. Rheology and Swelling Behavior

The cryogel specimens were sectioned into discs of identical sizes and equilibrated with deionized water. To assess their swelling properties, the relative water content in macropores known as capillary water (CW) and polymer-bound water (PW) were calculated using the following formulas: CW_%_ = (m_1_ − m_2_) / m_1_ × 100% and PW_%_ = (m_2_ − m_0_) / m_1_ × 100%, where m_1_, m_2_, and m_0_ are, respectively, the mass of fully hydrated, partially hydrated (after removal of weakly bound water or CW), and completely dried (heated in a thermostat at 90 °C) cryogel materials.

Rheological measurements of the swollen cryogels were carried out on an MCR 302 rotational rheometer (Anton Paar, Graz, Austria) at 37 °C. The frequency dependences of the elastic modulus (G′) and loss modulus (G″) were determined using the oscillation mode within the linear viscoelastic region (strain γ = 0.5%).

#### 2.4.4. Laser Scanning Confocal Microscopy (LSCM)

The incorporation and distribution of pyrene-conjugated IKVAV in the cryogels were analyzed by mapping the fluorescence intensity profile using laser scanning confocal microscopy (LSCM) on an LSM 780 microscope (Carl Zeiss). Zeiss Zen Black Software (2012) was used for acquisition.

### 2.5. Study of Condiut Constituents In Vitro

#### 2.5.1. Cell Culture

PC-12 cells and human skin fibroblasts (HSFs) were cultured aseptically in alpha-MEM supplemented with 10% fetal bovine serum (FBS), 2 mM L-glutamine, 100 U/mL penicillin, and 100 μg/mL streptomycin at 37 °C in a humidified air atmosphere with 5% CO_2_.

The cryogel samples were cut into round pieces, placed in a 24-well plate, and equilibrated with full medium for 1 h. The PC-12 cells were seeded on the top of the cryogels (6.4 × 10^4^ cells/cm^2^) and cultured for 72 h under standard conditions.

#### 2.5.2. Assessment of Cell Behavior in Cryogels

The cryogels with grown cells were transferred into new wells containing a fresh culture medium supplemented with MTS/PMS reagents to assess the cell viability/proliferation. Following a 1 h incubation in CO_2_-incubator (37 °C), optical absorbance of the generated formazan product was detected at 490 nm on an Infinite M200 PRO microplate analyzer (Tecan, Seestrasse, Switzerland) as a measure of the number of viable cells.

For bright-field microscopy analysis, the cryogels with cultured PC-12 cells were fixed with 4% p-formaldehyde for 1.5 h and gently washed with PBS. The fixed cells were subsequently stained with cresyl violet (0.1% *w/v* in ultrapure water) for 5 min and visualized using an AxioObserver Z1 microscope (Carl Zeiss, Oberkochen, Germany).

For LSCM analysis, the Gel-2 cryogel with cultured PC-12 cells was incubated with pre-warmed CellTracker Red dye (3.8 µg/mL in serum free medium) for 45 min under growth conditions, washed gently with HBSS, and additionally stained with bisBenzimidine trihydrochloride H33342 (5 µg/mL in PBS) for 10 min. The stained matrices were washed and fixed with 4% p-formaldehyde for 1.5 h and visualized using an LSM 780 microscope (Carl Zeiss).

#### 2.5.3. Verification of Compatibility with HSF

The HSFs were seeded on the top of the Gel-2 cryogel (10 × 10^4^ cells/cm^2^) and cultured for 72 h prior to cell detection with the MTS assay and bright-field microscopy as detailed in Section 2.5.2. To ensure lack of cytotoxicity of PCL-based tube, the PCL film 1 × 1 cm was aseptically incubated in 1 mL of full DMEM at 37 °C for 24 h to wash out soluble components. The conditioned medium was then added at different dilutions to pre-seeded HSFs, and the cells were cultured for 72 h followed by the MTS assay.

### 2.6. Study of Composite Conduit In Vivo

#### 2.6.1. Animals

Wistar male rats were purchased from NPK Biotech (Moscow, Russia). Animal care was performed according to the European regulations on the protection of experimental animals [Directive 2010/63/EU]. The animals were maintained under recommended temperature and humidity with free access to water and full-ration certified feed. The selected rats (350 ± 35 g) were divided into experimental groups (n = 8).

#### 2.6.2. Sciatic Nerve Diastasis Model

The animals were anesthetized by intraperitoneal injection of tiletamine–zolazepam/xylazine (20–20/10 mg/kg) and subjected to a conventional surgery to access the sciatic nerve at the left paw as detailed earlier [34]. The surgical manipulations were performed under an operating microscope. At the mid-thigh level, a 5 mm segment of the nerve was dissected with microsurgical scissors using a scale bar placed alongside the nerve. A hollow PCL-based tube or the composite conduit (both 2.5 mm in diameter and 5 mm in length) were decontaminated by keeping them in concentrated penicillin/streptomycin solution and washing with sterile isotonic solution. The materials were surgically connected to both stumps of the dissected nerve by 2–3 stitches using non-degradable Prolene sutures. Ketorolac (15 mg/kg) was additionally administered during the first 3 days post-implantation to reduce the pain syndrome. The treated animals were daily examined with respect to general state and state of the denervated paw.

#### 2.6.3. Electrophysiological Assessment

To assess neuromuscular function in the distal part of damaged sciatic nerve, the parameters of the motor response (M-response) of the muscle to electrical stimulation were analysed. Stimulating needle electrodes were inserted transcutaneously based on the projection area of the sciatic nerve. The stimulus intensity varied from 0.05 to 15 V, and the stimulus duration was 0.5 ms. A Model 1700 Differential AC Amplifier (A-M Systems, Sequim, WA, USA) was used to apply the stimulus, amplify, and record the responses. Changes in the maximum amplitude and thresholds of the evoked M-response were analyzed.

#### 2.6.4. Histological Analysis

Following the in vivo experiment, the animals were sacrificed, and the treated nerves with the surrounding tissues/conduits were dissected post mortem. The explants were subjected to chemical fixation and treatment with organic solution and finally were imbedded in paraffin medium according to the established recommendations [35]. The transverse and longitudinal sections of the distal part of the nerve in contact with the conduit were cut on a Microm HM355 microtome (Thermo Scientific). The sections were subjected to histological staining [35] and visualized by bright-field microscopy under an Axio Observer Z1 microscope (Carl Zeiss).

### 2.7. Statistical Analysis

The data were presented as mean ± standard deviation (SD) or standard error (SEM). Statistical significance was determined using the Student’s *t* test (* *p* < 0.05) and the one-way analysis of variance (ANOVA) followed by Tukey’s multiple comparison post-test (* *p* < 0.05, ** *p* < 0.01, *** *p* < 0.001).

## 3. Results

### 3.1. Design of Composite Cryogel

**Figure 1:**
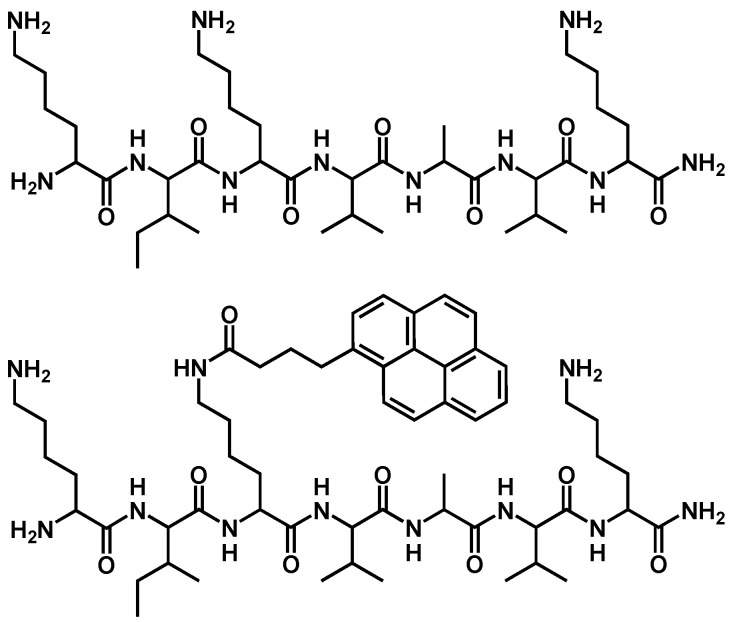
Structure of KIKVAVK peptide (**1**) and its fluorescent derivative (**2**).

Gelatin was used as a gel-forming proteinaceous macromer, which can be polymerized by different chemical methods such as cross-linking with glutaric aldehyde and other bifunctional agents towards amino groups [36]. It was supposed that additional derivatization of an ECM peptide with terminal lysine residues from both peptide ends would allow its in situ inclusion into the cross-linked protein network to produce a composite protein–ECM peptide hydrogel (cryogel). For this purpose, KIKVAVK analog of IKVAV laminin-derived peptide (1) and its conjugate with pyrene label (2) were synthesized using SPPS technique with Fmoc-protected amino acids (Figure 1). The synthesized products were chromatographically homogeneous peptides with characteristic m/z values, namely, 392.79 [M + 2H]^2+^, 784.58 [M + H]^+^, 806.56 [M + Na]^+^, (1) and 1054.68 [M + H]^+^, 1076.67 [M + Na]^+^ (2) (Supplementary Materials).

Gelatin and KIKVAVK were cross-linked at final concentration of the peptide of 0, 0.025, 0.1, 0.4 wt.% under cryotropic gelation conditions. The corresponding cryogels were denoted as Gel-0, Gel-1, Gel-2, and Gel-3, respectively.

### 3.2. Characterization of Composite Cryogels

**Figure 2:**
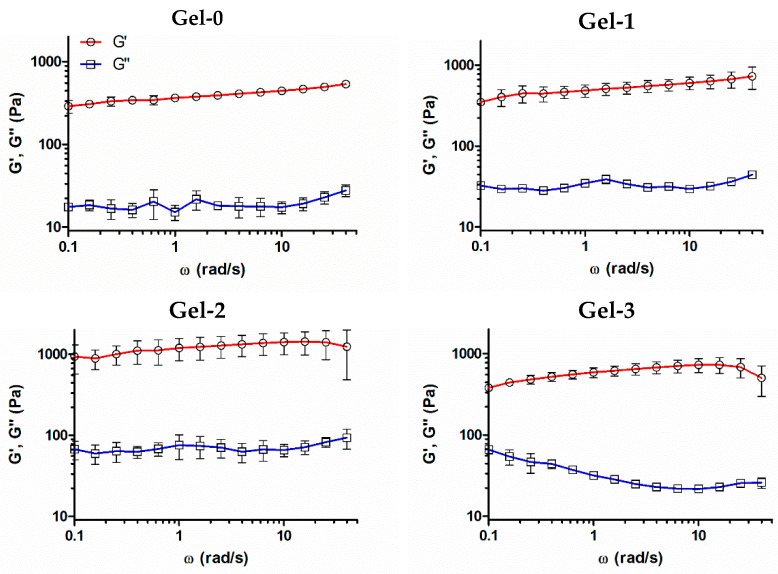
Frequency sweep analysis of storage modulus (G′) and loss modulus (G″) of cryogels with different amounts of peptide component (0–0.4 wt.%). Measurements were performed within LVR at γ = 0.5% shear strain deformation. The data are presented as mean ± SD.

Viscoelastic properties of the cryogels were analyzed using small-amplitude oscillatory shear measurements at a strain amplitude (γ) of 0.5%. The storage modulus (G′) and the loss modulus (G″) of the materials showed weak-to-moderate dependence on angular frequency in the range up to ω = 40 rad s^−1^ (Figure 2). The formation of a well-structured hydrogel network with a dominant elastic behavior was confirmed (G′ >> G″). Both moduli increased in proportion with the peptide amount in Gel-0, Gel-1, and Gel-2, supporting incorporation of the peptide molecules into the cryogels. This was accompanied by some decrease in the G′/G″ ratio of the materials as if the peptide component modulated the hydrogel network, which preserved elastic nature. The G′ and G″ moduli of the tested samples did not cross with the increase in the angular frequency and were practically independent of it in the studied range, demonstrating the formation of a stable well-structured hydrogel network with a dominant elastic behavior [37,38]. Further increase of the peptide amount to 0.4 wt.% in Gel-3 weakly contributed to G′ augmentation and distorted the frequency dependence of G″, presumably due to a decrease in the material homogeneity (Figure 2).

**Figure 3:**
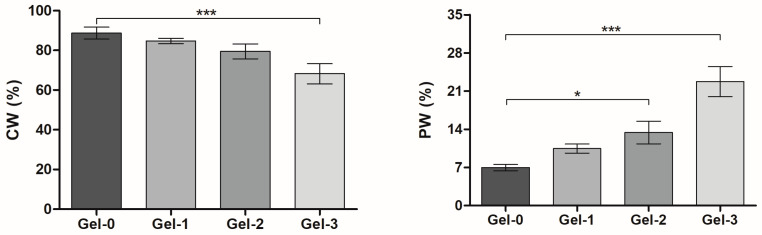
Swelling properties of cryogels with different amounts of peptide component (0–0.4 wt.%). Relative percentage of capillary water (CW) and polymer-bound water (PW) was shown (mean ± SD, * *p* < 0.05, *** *p* < 0.001).

As previously shown, differentiation of water content in macropores (capillary water, CW) and the polymer network (polymer-bound water, PW) serve as a parameter to characterize the structure of the cryogels and effect of their constituents [39]. The gradual decrease in CW content in Gel-0, Gel-1, and Gel-2 from 89 to 80% along with increase in PW from 7 to 13.4% were observed (Figure 3), suggesting some reduction of macropore size due to additional swelling of the polymer component mixed with peptide molecules. Further non-linear change of the detected parameters in Gel-3 (Figure 3) is explained by a certain structure transition in the mixed hydrogel system in agreement with viscoelastic behavior (Figure 2).

**Figure 4:**
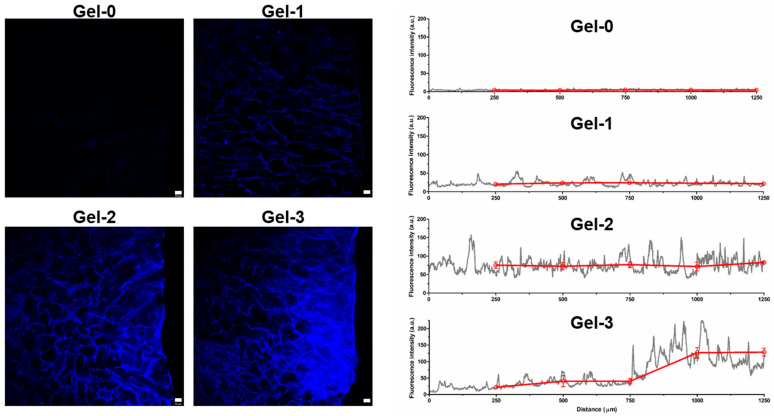
LSCM images of bulk sections of cryogels with different amounts of pyrene-labeled peptide (0–0.4 wt.%) and fluorescence intensity profiles along these sections (red curve represents the average fluorescence profile for each 250 μm distance, mean ± SD). Scale bar is 50 µm.

LSCM analysis of cut sections of the cryogels containing the labelled peptide additionally supported homogeneous modification of the materials with peptide molecules in a proportional manner for Gels 1 and 2, whereas at increased peptide amount in Gel-3, such a homogeneity impaired presumably due to non-uniform distribution of the peptide component in the cross-linked polymer network (Figure 4).

### 3.3. Cell Behavior in Composite Cryogels

#### 3.3.1. Growth and Distribution

**Figure 5:**
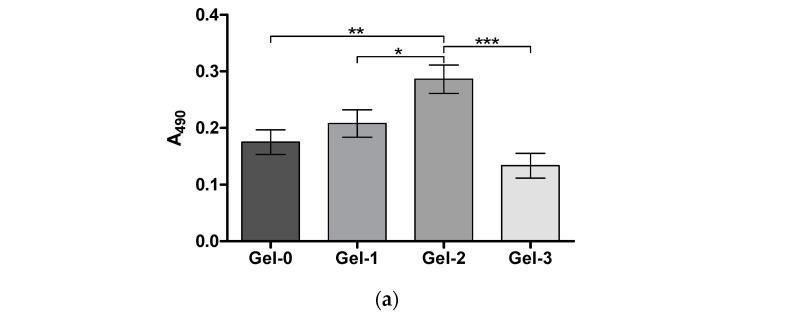

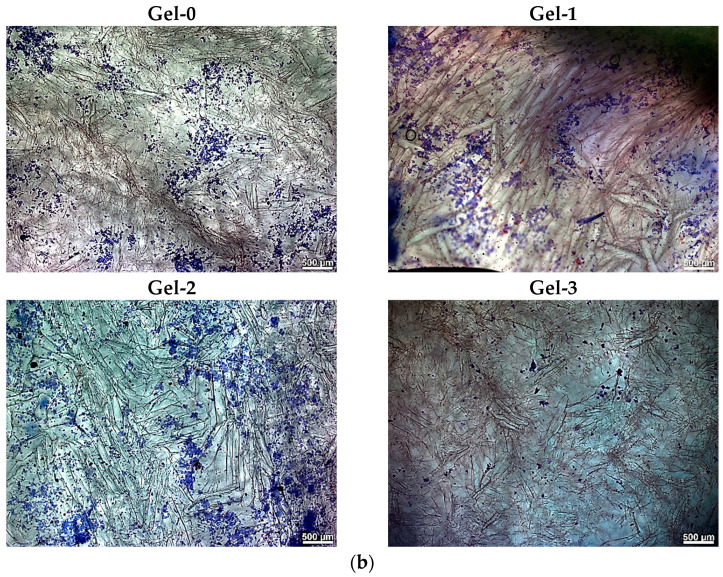
(**a**) Proliferation (viability) of PC-12 cells cultured in peptide-modified cryogels according to MTS assay (72 h, mean ± SD, * *p* < 0.05, ** *p* < 0.01, *** *p* < 0.001). (**b**) Representative bright-field microscopy images of PC-12 cells in the matrices after fixing and staining with cresyl violet.

After a 72 h culture of PC-12 cells in the cryogels, the detected MTS signal increased in the following order: Gel-0 < Gel-1 < Gel-2. As shown earlier, this signal generally correlates with cell number in different cryogel matrices [40,41]. The results suggest that the peptide component noticeably promotes cell proliferation up to 64% for 0.1 wt.% peptide (*** *p* < 0.001). At a higher concentration in Gel-3, the introduced peptide, however, tended to inhibit cell proliferation in comparison with the peptide-free material (Figure 5a).

These results were in agreement with the bright-field microscopy analysis of cresyl-violet stained cells used as a screening method to assess cell behavior in the cryogels [42]. An increased number of PC-12 cells in the peptide-containing cryogels (Gel-1 and Gel-2) was observed along with better cell distribution in these materials compared to the control. At the highest peptide concentration (Gel-3), the peptide’s effect on cell proliferation was reversed so that fewer cells were detected in the fields of view (Figure 5b).

Collectively, the results show that incorporation of KIKVAVK peptide into the cryogel up to a critical concentration (0.1 wt.%) allows enhanced cell distribution and growth over the peptide-free counterpart. At a higher concentration, peptide-mediated bioactivity can be impaired in relation to non-homogeneous distribution or other probable factors such as non-optimal ligand–receptor ratio [24].

#### 3.3.2. Morphological Differentiation

**Figure 6:**
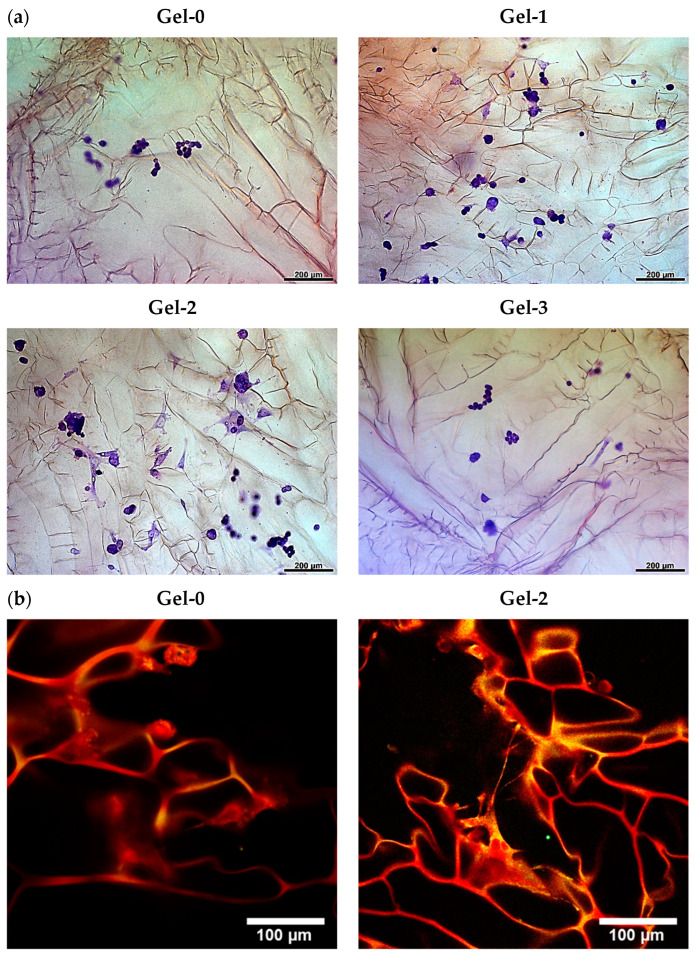

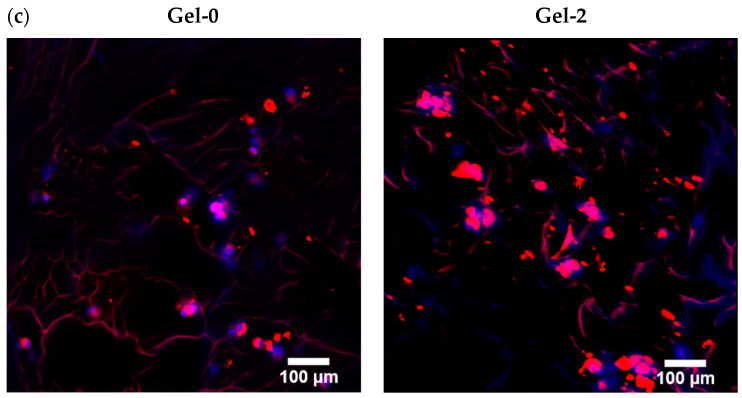
Morphology of PC-12 cells cultured for 120 h in the cryogels according to (**a**) bright-field microscopy and (**b**) LSCM of cresyl violet-stained cells and (**c**) LSCM of CellTracker Red/Hoechst 33342-stained cells.

The morphology of PC-12 cells grown in the cryogels was also assessed at 120 h (Figure 6a). In the control material, the cells mostly showed initial round morphology. The cells grown in the peptide-modified cryogels featured increased spreading and sprouting attributed to the induction of differentiation process into neuron-like cells. Such a morphological differentiation was generally in proportion to the mitogenic effect (Figure 5). In particular, Gel-2 better supported morphological alterations in the cells, which acquired extended sprouts generally up to 5 sprouts with a length of up to 200 µm (Figure 6a).

Additional LSCM analysis of the PC-12 cells in Gel-2 stained with CellTracker Red and cresyl violet confirmed this observation, showing a rearranged cell morphology in contact with the material (Figure 6b). Together, these results suggest the in vitro neurogenic effect of the peptide component in the cryogel matrix in a dose-dependent manner.

### 3.4. Design and Characterization of Composite Conduit

This cryogel was further used to prepare a prototype of the nerve conduit. Since soft non-reinforced hydrogels are commonly not compliant with surgical suturing, a conduit shell in the form of a tube was additionally prepared. Poly-ε-caprolatone (PCL) was selected as the main constituent of the tube, considering its wide use to produce firm and elastic biomaterials including resorbable sutures [43,44]. PCL was mixed with lecithin, considering improving effects of this additive on the biocompatibility and elasticity of electrospun PCL-based scaffolds [45,46]. PCL-lecithin film was prepared by the solvent evaporation method and assessed in comparison with one-component PCL counterpart (Figure S2). The presence of lecithin component was confirmed by FTIR spectroscopy, which showed characteristic lecithin bands of symmetric CH_2_ stretching at 2853 cm^–1^ and asymmetric CH_2_ stretching at 2923 and 3009 cm^−1^, respectively [39]. The FTIR spectra of different areas of the PCL-lecithin material were the same, indicating the formation of homogeneous film. According to DSC analysis, the PCL film generated a single endothermic peak at ca. 58 °C, which corresponds to its melting point. In the case of PCL-lecithin film, this value was slightly reduced to 56 °C, and wide low-intensity peaks at ca. 200 and 250 °C appeared (Figure S2), reflecting interaction of PCL with the additive [47,48].

Pre-formed PCL-lecithin films were used to prepare conduit shell tubes with a diameter of 2.5 mm, which generally matches the rat sciatic nerve in the area of diastasis. Considering mechanical behavior as an important factor for conduit performance upon PN regeneration, the tensile strength of these tubes was estimated by measuring the maximum pull force before complete tensile failure upon lengthwise stretching. The tubes were destroyed averagely at 6.0 ± 1.2 N, which is moderately above the reported value for a rat’s sciatic nerve, namely, 5.5 ± 0.5 N [49].

**Figure 7:**
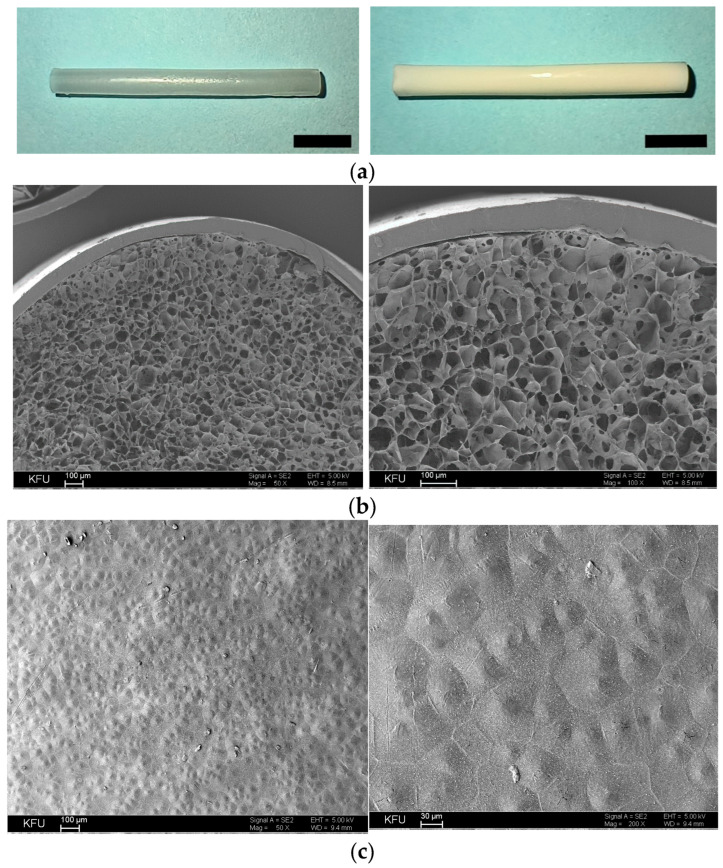
(**a**) Photographs of hollow polymer tube (**left**) and composite conduit (**right**) 2.5 mm in diameter and 5 mm in length (scale bar is 1 mm). (**b**) SEM images of cross-section of the composite conduit. (**c**) SEM images of the surface of polymer tube at different magnifications.

Furthermore, composite cryogel (Gel-2 composition) was formed inside the polymer tube to produce the composite conduit of 5 mm in length (Figure 7a) to fit the size of introduced sciatic nerve gap in vivo. SEM images of the conduit cross-section (Figure 7b) and the surface of polymer tube (Figure 7c) at different magnifications show that Gel-2 forms a homogeneous porous scaffold inside the tube, whereas the tube has a uniform thickness of ca. 82 µm and a relatively rough but homogeneous surface.

Prior in vivo study, the in vitro biocompatibility of the developed NC was verified using primary human skin fibroblasts (HSF). The NC constituents including Gel-2 composition and the PCL-based tube did not decrease proliferation and viability of HSFs, confirming their cytocompatible properties (Figure S3).

### 3.5. Neuroregenerative Effects of Composite Conduit

#### 3.5.1. In Vivo Behavior of Implanted Materials

**Figure 8:**
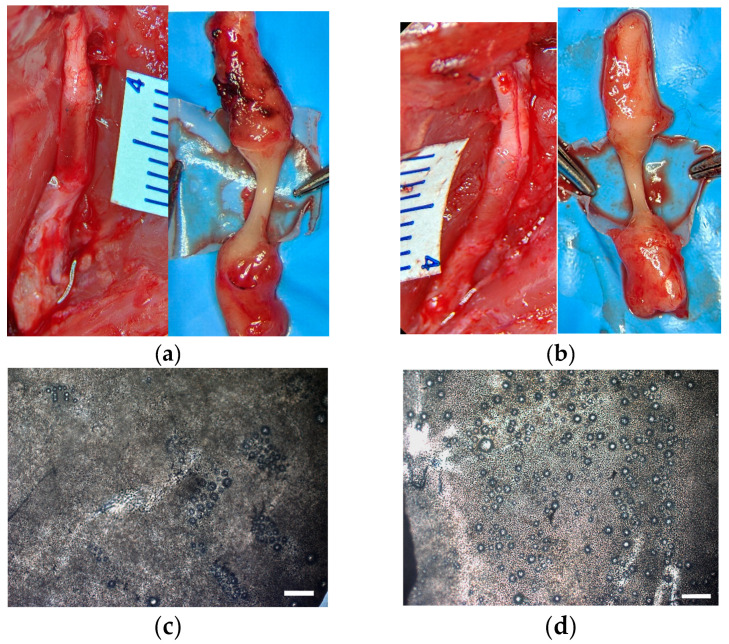
Representative photographs of (**a**) implanted hollow polymer tube and (**b**) composite conduit sutured to sciatic nerve stumps as well as interior of the materials 8 weeks post-implantation. Bright-field microscopy images of tube surface: (**c**) hollow tube and (**d**) composite conduit. Scale bar is 500 µm.

The hollow polymer tube filled with isotonic solution (group 1) and the composite conduit filled with peptide-modified cryogel (group 2) were studied and compared using sciatic nerve diastasis model. The materials were implanted into 5 mm diastasis as detailed in Section 2.6.2, and their in vivo effects were evaluated during 8 weeks. The polymer tube was compliant to surgical suturing in agreement with its sufficient tensile strength. It was handy to be sewed to the epineurium of nerve stumps and preserved its integrity and localization in both groups during the whole experiment, confirming firm attachment of the tube to damaged nerve (Figure 8a,b).

Ingrowth of new tissue inside the tube (in the area of diastasis) was observed in both groups with no signs of neuroma formation. Furthermore, no residues of the implanted cryogel were detected, indicating complete resorption of the filling material (Figure 8a,b). Although the tube did not disintegrate, it became more fragile and transparent with a number of round holes tens micron in diameter (Figure 8c,d), attributed to biodegradation of the PCL-based shell [50]. Interestingly, such a biodegradation was more profound in group 2 compared to group 1. This can be explained by the ability of cryogel to promote cell propagation inside the tube, thus additionally supplying enzymes involved in PCL hydrolysis.

#### 3.5.2. Electromyography Study

**Figure 9:**
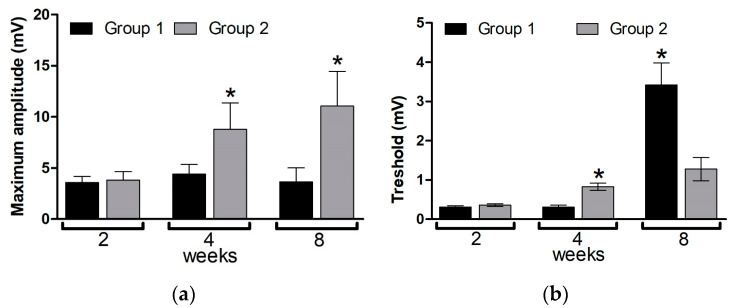
(**a**) Maximum amplitude and (**b**) threshold of evoked M-response in damaged sciatic nerve. Mean ± SEM are shown, * *p* < 0.05.

Figure 9 shows parameters of the motor response (M-response) upon electrical stimulation of damaged sciatic nerve in dynamics. The maximum amplitude is a measure of motor unit involvement in muscle contraction, and it profoundly drops after nerve dissection [51]. At week 2, this signal was similarly diminished in both groups compared. Afterwards, it significantly increased at 4 and further at 8 weeks only in the conduit group, whereas no significant recovery of the amplitude occurred in the hollow tube group during the observation period (Figure 9a).

In addition, we analyzed the threshold of evoked M-response that corresponds to the stimulus intensity at which the most sensitive motor neurons are activated. The moderate increase in this signal at 4 and 8 weeks detected in group 2 (Figure 9b) along with the amplitude restoration (Figure 9a) suggest the course of a regeneration process [51]. Unlike such an effect of the conduit, the hollow tube showed excessive elevation of the threshold value at 8 weeks (Figure 9b) without increase of the maximum amplitude (Figure 9a), indicating inhibition of motor neuron activity in the damaged nerve. These data suggest that the composite cryogel inside the conduit improves signal transduction in the damaged nerve and therefore imparts regenerative activity to the developed NC.

#### 3.5.3. Histological Study

To characterize the tissue repair process at the distal part of damaged nerve, histological analysis was performed at 8 weeks. Cross-sections of the nerve were assessed by three types of staining, namely, phenothiazine dye-based Giemsa staining for visualization of cells and ECM, luxol fast blue/cresyl violet for myelinated structures and Schwann cells, and Bielschowsky silver-based staining for axonal structures [50,52].

**Figure 10:**
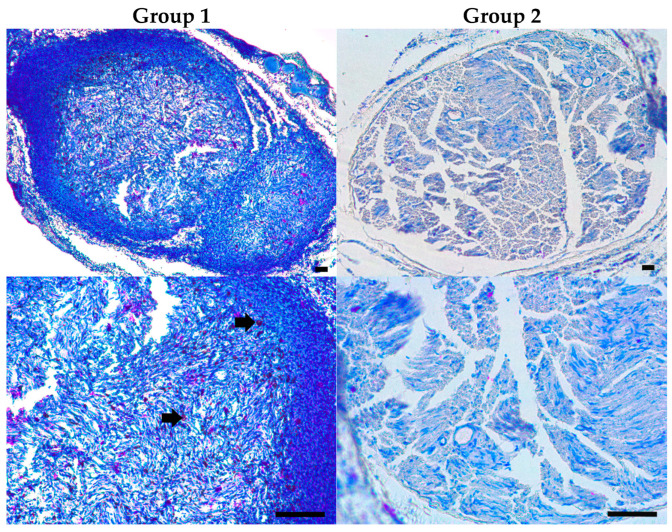
Typical bright-field images of cross-section of treated sciatic nerve stained by Giemsa method. Group 1—hollow tube, Group 2—composite conduit (8 weeks). **Upper** and **lower** panels show the same samples at different magnifications (scale bar is 200 µm).

According to the first staining (Figure 10), group 1 showed intensively stained blue tissues attributed to a weakly structured non-myelinated matrix containing a number of Schwann cells and macrophages (large violet). These cells are involved in posttraumatic degradation and resorption of nerve tissues (Wallerian degeneration), which accompany the repair process. Degenerating axonal fragments were detected as scattered dark-blue structures (Figure 10, arrows). Such a morphological pattern suggests slow regeneration process [53,54], which occurs in the absence of a supporting matrix in the diastasis area.

In group 2, the detected tissues were weakly stained in blue and contained fewer cells (Figure 10), indicating a later regeneration phase such as proliferation/remodeling [53,54].

**Figure 11:**
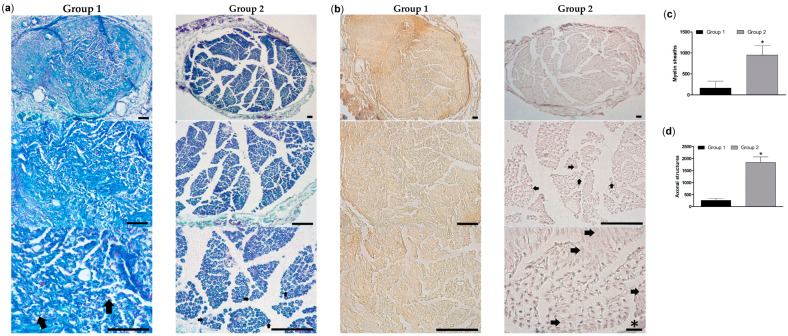
Typical bright-field images of cross-section of treated sciatic nerve stained with (**a**) luxol fast blue/cresyl violet and (**b**) by Bielschowsky method. Group 1—hollow tube, Group 2—composite conduit (8 weeks). Three images at different magnifications for each group were presented; scale bar is 200 μm except the bar with asterisk (20 μm). (**c**) Number of myelin sheaths per section detected in (**a**). (**d**) Number of axonal structures per section detected in (**b**). Mean ± SEM are shown, * *p* < 0.05.

According to the second staining, compared to group 2, group 1 featured a less intensively stained and less structured matrix with a number of separate crescent-shaped Schwann cells (Figure 11a, arrows) and few defined myelin sheaths. Degenerating axons also appeared in this group. In group 2, a lot of mature and forming myelin sheaths (arrows) were observed, and most of the detected Schwann cells were associated with these structures (Figure 11a,c).

According to the third staining, images in the compared groups exhibited different coloring attributed to proteinaceous tissue (group 1) and lipidous (myelinated) tissue (group 2) [52] (Figure 11b). In the latter matrix, there were many axonal structures of different maturity (Figure 11b,d).

In addition, hematoxylin-eosin (H&E)-stained longitudinal sections of the diastasis area further supported different repair processes in the groups. In the case of the hollow tube, non-oriented connective tissue was generally observed with a number of infiltrated cells and a lack of nerve fibers, indicating a prolonged inflammation process and delayed regeneration. In contrast to this, the cryogel scaffold promoted the formation of oriented fiber structures, which penetrated into the distal part of degrading nerve (Figure S4).

Altogether, our study shows that the composite conduit profoundly accelerates sciatic nerve repair in comparison with the hollow tube counterpart. This proves the regenerative action of peptide-activated cryogel in vivo, in addition to in vitro behavior, and also suggests the therapeutic potential of the proposed construct in treating PN lesions.

## 4. Discussion

Numerous designs of engineered materials for neural tissue repair have been proposed using different polymeric constituents and fabrication and application principles. The therapeutic efficacy of such biomaterials was recognized to be associated with the presence of active ingredients that promote regenerative events in damaged nerves [55,56]. In spite of the wide use of macroporous cryogels as an advanced hydrogel ‘platform’ for tissue engineering [57], relatively few cryogel scaffolds for both peripheral and central nervous systems have been proposed to date [18,19,22,58,59]. A combination of cryogels with polypeptides/proteins with cytoadhesive and signaling properties such as ECM proteins and growth factors (GFs) was shown to be a promising strategy for neuroregeneration [22,59]. For instance, cryogels formed by gelatin–laminin and dextran–laminin compositions showed neurogenic activity toward human cord blood-derived stem cells in vitro as well as in contact with brain tissues ex vivo and in vivo [59]. Aligned chitosan–gelatin cryogel-based conduit with pre-adsorbed NGF enhanced regeneration of a critical sciatic nerve lesion in rats [22].

The medical use of biotechnologically produced macromolecules in engineered materials is complicated by their low stability, increased cost, and immunogenic properties. Although small synthetic peptides have proved to be effective mimetics of ECM proteins and GFs and more compliant to combination with scaffolds [25,60,61], there is still a lack of studies on such oligopeptide-based cryogels. The existing related designs that are based on peptide systems refer to non-spongious hydrogels and self-assembling nanofiber hydrogels.

Several ECM-derived peptide motifs were combined with hydrogel materials to improve neuroregeneration. In particular, YIGSR was adsorbed on a polyacrylamide/graphene oxide/gelatin/sodium alginate composite conduit by a simple soaking process [62], whereas IKVAV or YIGSR acrylate derivatives were incorporated into poly (lactide ethylene oxide fumarate) [24] and poly (ethylene glycol) methacrylate [63] hydrogels by a radical polymerization method. In addition, CSRARKQAASIKVAVSADR peptide was pre-linked to silk fibroin by a carbodiimide method to further form an IKVAV-modified matrix for NSCs [27].

More commonly, neurogenic peptides are introduced as a part of self-assembling peptides (SAPs), which form biomimeting nanofiber scaffolds. Previously, in RADA_16_-based scaffolds, YIGSR moiety increased the viability and differentiation of NSCs [64]; bone marrow homing peptide 2 (BMHP2) and RGD composition promoted differentiation of NSCs, whereas the single peptides had a mitogenic effect [65]. IKVAV epitopes in SAP hydrogel suppressed the astrocytic differentiation of neural progenitor cells and inhibited glial scar formation [66,67]. X. Wang et al. developed a series of RADA-based hydrogels for PN repair functionalized with a combination of GF- and ECM-mimeting peptides such as BDNF-derived RGIDKRHWNSQ (RGI) and VEGF-derived KLTWQELYQLKYKGI (KLT) [60]; RGI and NGF-derived CTDIKGKCTGACDGKQC (CTD) [25]; IKVAV and RGI [68] with proved regenerative activities of single peptide moieties and synergistic/additive effects of their compositions in 10 mm sciatic nerve defect in rats.

Direct cross-linking of an ECM oligopeptide sequence having additional reactive lysine residues with the protein cryogel proposed in our study can be regarded as an alternative approach to the aforementioned designs for scaffold production. It is beneficial compared to the reversible adsorption process and less laborious compared to SAP technology. Although the reaction with internal lysine residue of IKVAV motif could not be completely avoidable, it should not result in the loss of its bioactivity or even more so in the antagonistic action according to our data. This approach resembles the earlier reported design based on the copolymerization of CCRRIKVAVWLC having terminal cysteine residues with multi-arm thiolated PEG to form model hydrogel matrix via disulfide linking [69]. Nonetheless, the IKVAV-containing proteinaceous cryogel we have developed is a part of a composite medical device. In particular, pre-synthesized KIKVAVK was controllably incorporated into the gelatin-based cryogel by chemical cross-linking as supported by rheological, swelling, and LSCM data (Figure 2, Figure 3 and Figure 4) as well as by cellular responses in the matrix (Figure 5 and Figure 6).

Importantly, our data show that the linked peptide molecules preserve bioactivity according to their effects on cell proliferation and morphological differentiation, additionally suggesting the existence of an optimal density range for the peptide motif (Figure 5 and Figure 6). In agreement with this, the adhesive and proliferative effects of CCRRIKVAVWLC peptide at a concentration of 10 μM in PEG hydrogel were reported to be higher than those at 50 and 100 μM [69]. Furthermore, mouse embryonic stem cells grown in PEG hydrogel modified with a gradient of IKVAV exhibited increased neurogenic differentiation at an intermediate peptide concentration of 60 μM [63]. Excess peptide concentrations may be oversaturating, whereas low to intermediate concentrations should activate cellular pathways [63]. In our study, an IKVAV concentration of 0.1 wt.%, which corresponds to a density in the composite cryogel of ca. 10 µg/cm^3^, was found to be more responsive in vitro, and therefore it was selected for in vivo study. The use of cryogel scaffold with an interconnected macroporous network does not require the creation of a peptide gradient, which is commonly implemented in conventional hydrogel materials to support bulk migration of neural cells [70,71]. Moreover, a homogenous distribution of peptide molecules (Figure 4, Gel-2) should favor cell–matrix interactions and promote cell adherence, proliferation, and differentiation.

The combination of the developed cryogel with a PCL-based shell tube was essential in our design to surgically attach the conduit to nerve stumps and expectedly to withstand tension and prevent the collapse of the inner matrix in accordance with previous observations [5]. Interestingly, there are reports on using quite soft hydrogel conduits per se for the same application; however, details of their stability upon implantation are often not provided [18,19].

Several polymer tubes both hollow and filled with biomaterials were assessed in PN damage models. For instance, a polyurethane-based tube filled with an aligned chitosan–gelatin cryogel was prepared and tested ex vivo using dorsal root ganglion culture [21]. A permeable electrospun poly (lactide-co-ε-caprolactone) (PLCL) membrane with 3D-printed gelatin-based paths was used to construct the NC, which promoted axonal regeneration, remyelination, and functional recovery of an 8 mm sciatic nerve gap [34]. This design was recently extended to develop an electrospun PLCL-silk fibroin-NGF blend conduit with interior tannic acid-polypyrrole-RGD composite hydrogel with significant therapeutic potential [72]. Similarly, an electrospun PCL conduit was combined with a methacrylated gelatin hydrogel bearing CNTF/IGF-1 GFs [73] or modified with RGD and YIGSR peptide derivatives bearing naphthalene and diphenylalanine moieties, which self-assemble on the material’s surface [23]. The latter conduit had an inner diameter of 1.5 mm as well as a wall thickness of 550 µm, which is significantly bigger compared to the PCL shell obtained in our study. Using photo-cross-linkable PCL derivative, a 3D-printed cylindrical NC loaded with NGF and filled with an aligned gelatin cryogel scaffold was developed [22].

Our in vivo results confirmed the neurogenic activity of the IKVAV-containing NC with a PCL-based shell tube in comparison with the hollow tube counterpart upon implantation into 5 mm sciatic nerve diastasis in rats. Smaller size defects were also reported [62,74] as well as more critical ones up to 15 mm in length [19,22,25,60,68]. According to surgical assessment, the PCL-based tube per se possessed sufficient persistence to be stably sutured to the nerve and sustain long-term implantation (Figure 8). Visualization of the trauma at 8-week period post-implantation showed complete resorption of the cryogel matrix as well as the course of resorption process in the polymer shell (Figure 8), which is expected to completely degrade eventually.

The ability of the developed cryogel to promote PN repair was assessed by consistent electrophysiological and morphological data (Figure 9, Figure 10 and Figure 11 and Figure S4). This appeared in the absence of the lumens, channels, or matrix alignments introduced in some previous cryogel designs [19,20], suggesting that nerve fibers can regenerate along the entire cryogel scaffold, probably in conjunction with its gradual degradation and replacement. Earlier, the therapeutic potential of one-component gelatin cryogel was demonstrated on examples of thick-wall conduits with a large lumen in the neurorrhaphy model when the material was wrapped around a pre-sutured sciatic nerve [18] as well as in the conventional diastasis model when the material was directly attached to the epineurium; in the latter study, the regenerative effect of the cryogel was enhanced by its combination with adipose-derived stem cells [75].

Due to lack of reported synthetic peptide-modified cryogel scaffolds for PN repair, the promoting effect of IKVAV-containing cryogel on functional and morphological recovery of the damaged sciatic nerve can be compared with the aforementioned SAP scaffolds bearing peptide motifs RGI, KLT, CTD, and IKVAV [25,68,76]. Analysis of the reported data show that although they deal with a more critical lesion of the sciatic nerve (10 mm gap), the myographic activity and histological profile of the myelinated structures revealed in these studies were generally inferior compared to our data (5 mm gap). Therefore, the results of this study encourage further investigation of the therapeutic potential of the developed composite conduit in comparison with commercial products and autografts.

## Data Availability

The original contributions presented in this study are included in the article/Supplementary Material, and further inquiries can be directed to the corresponding author.

## Supplementary Materials

The following supporting information can be downloaded at: https://www.mdpi.com/article/10.3390/pharmaceutics16121569/s1, Figure S1: (a) HPLC-chromatograms of KIKVAVK and KIK (pyrene)VAVK peptides with UV detecting (left) and fluorescent detecting (right). Mass spectrum of (b) KIKVAVK and (c) KIK (pyrene)VAVK peptides; Figure S2: (a) FTIR spectrum and (b) DSC thermograms of PCL and PCL-lecithin films; Figure S3: (a) Proliferation (viability) of HSF cultured in the medium pre-exposed to PCL-based film (MTS assay, 72 h, mean ± SD). (b) Proliferation (viability) of HSF cultured in peptide-modified cryogel (MTS assay, 72 h, mean ± SD). (c) Representative bright-field microscopy images of HSF 72 h post-seeding in the matrices; Figure S4: H&E-stained longitudinal sections of diastasis area (upper panel) and adjacent distal part of damaged sciatic nerve (lower panel) at different magnifications.
