# Supplementary material for: Newly Designed PCL-Wrapped Cryogel-Based Conduit Activated with IKVAV Peptide Derivative for Peripheral Nerve Repair
Source: Pharmaceutics. 2024 Dec 8;16(12):1569. doi: 10.3390/pharmaceutics16121569 (PMC11677967; doi:10.3390/pharmaceutics16121569)
Supplement: Supplementary file 1 [file pharmaceutics-16-01569-s001.zip › pharmaceutics-3290125-supplementary.pdf]

pharmaceutics

MDPI

Article

Supplementary Materials: Newly Designed PCL-Wrapped Cryogel-Based Conduit Activated with IKVAV Peptide Derivative for Peripheral Nerve Repair

Abdulla Yergeshov, Mohamed Zoughaib, Kenana Dayob, Marat Kamalov, Duong Luong, Albina Zakirova, Ruslan Mullin, Diana Salakhieva, Timur I. Abdullin

(a)

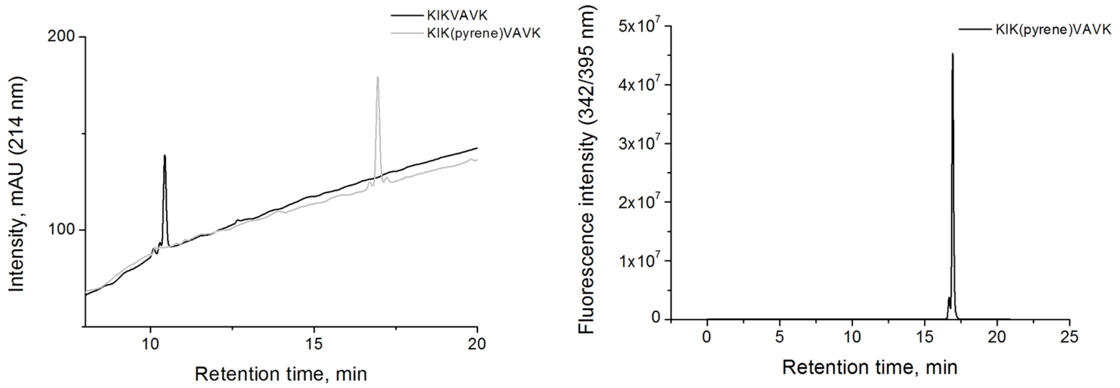

(b)

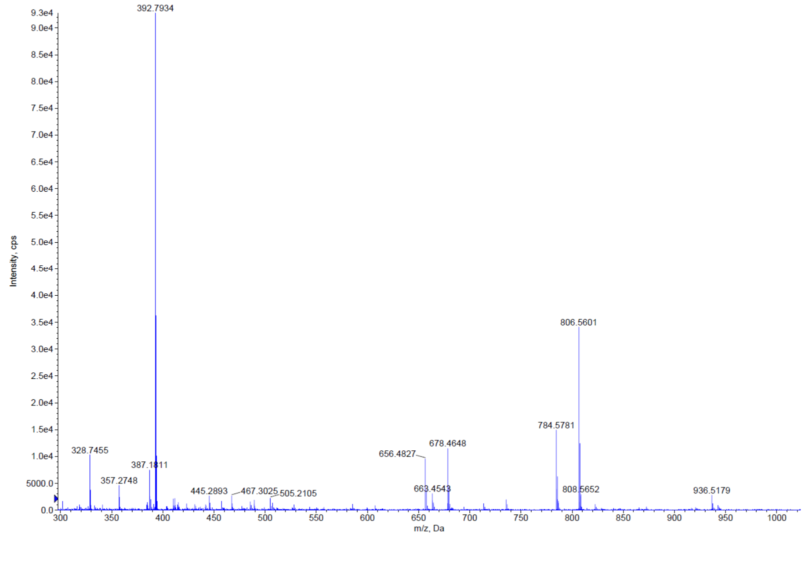

(c)

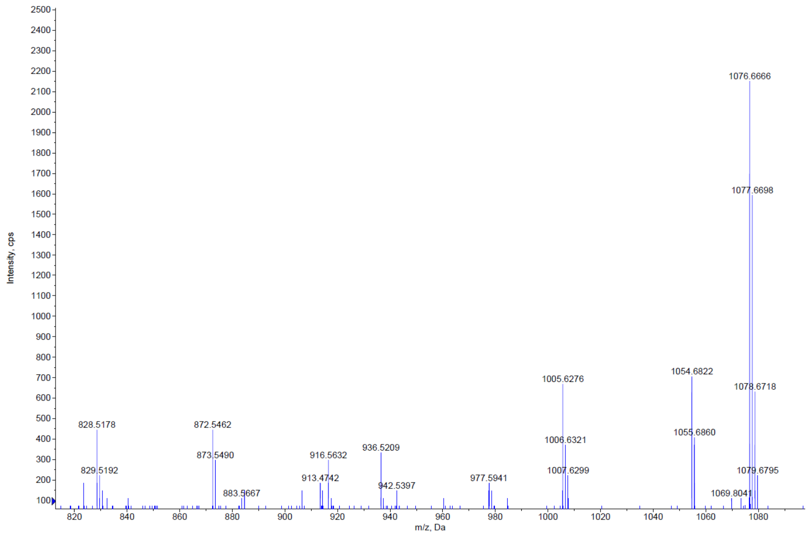

Figure S1. (**a**) HPLC-chromatograms of KIKVAVK and KIK(pyrene)VAVK peptides with UV detecting (left) and fluorescent detecting (right). Mass spectrum of (**b**) KIKVAVK and (**c**) KIK(pyrene)VAVK peptides.

.

(a)

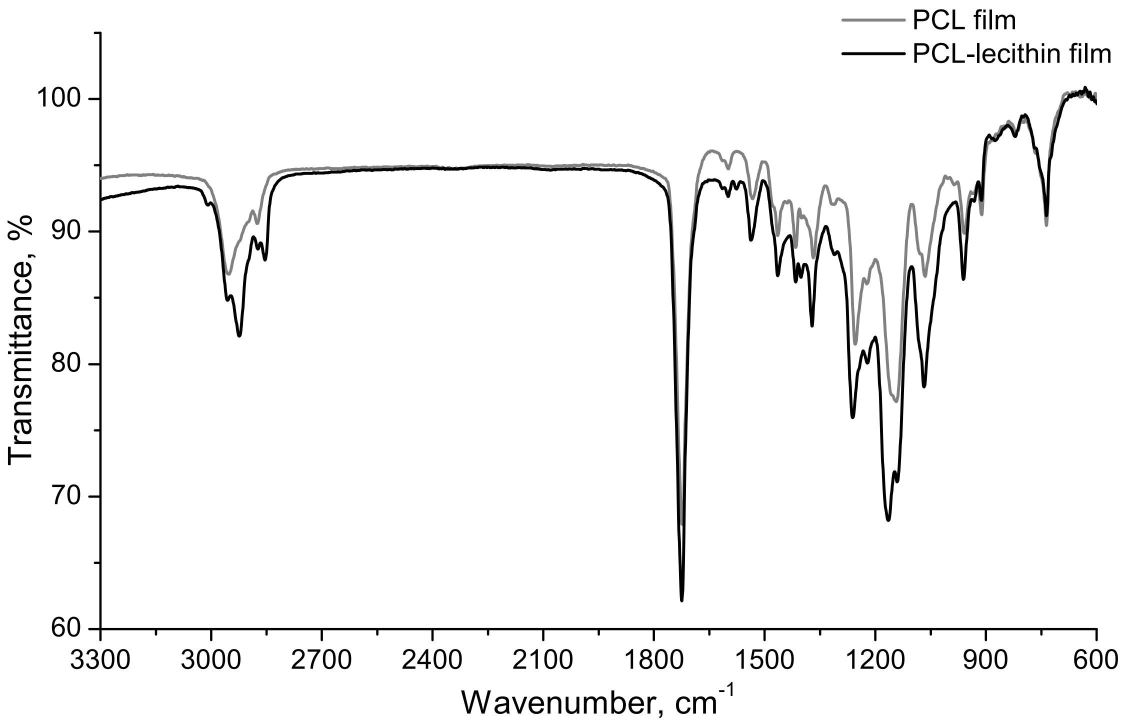

(b)

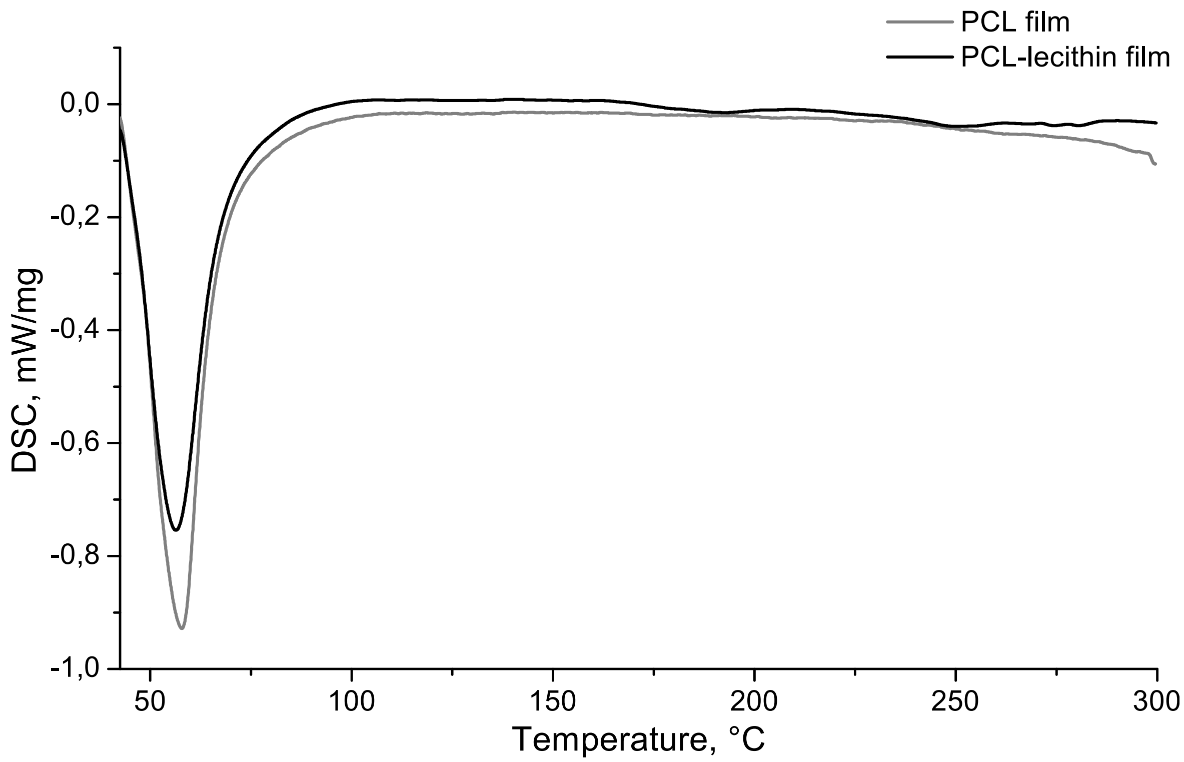

Figure S2. (**a**) FTIR spectrum and (**b**) DSC thermograms of PCL and PCL-lecithin films.

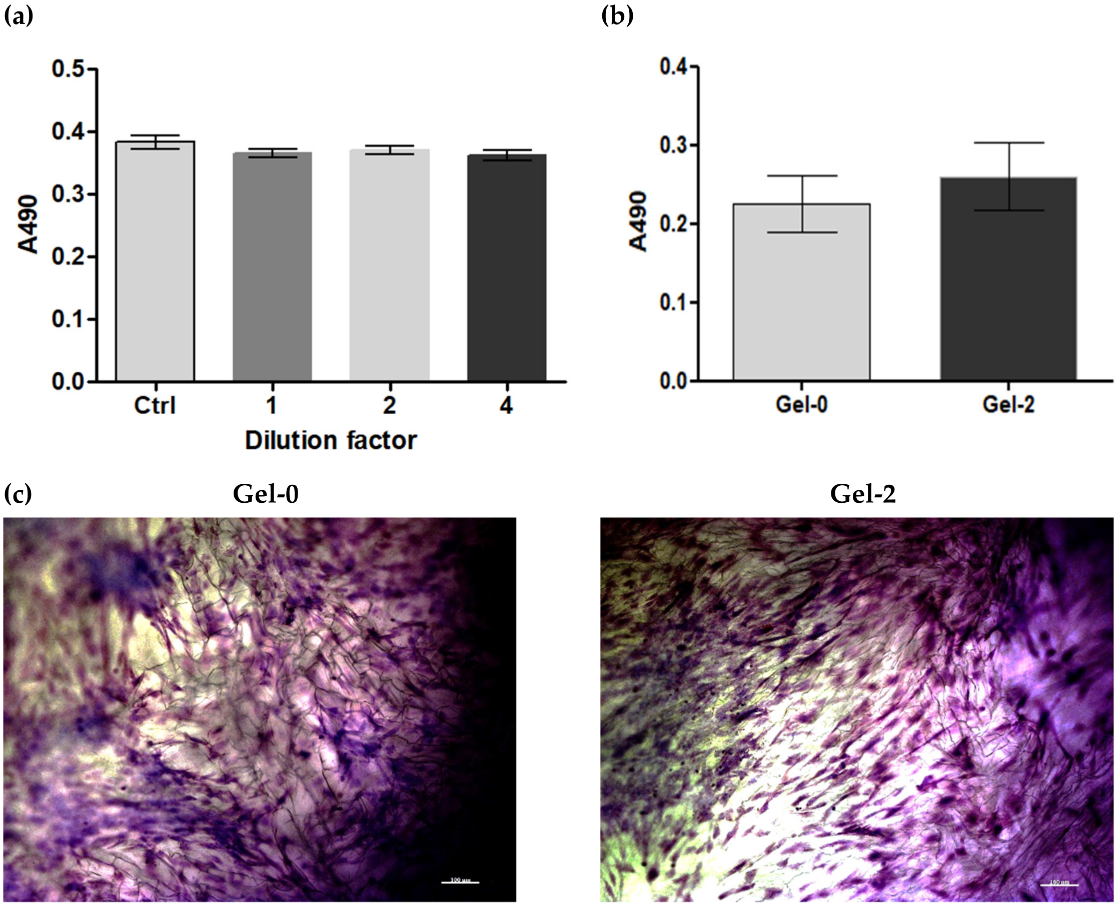

Figure S3. (**a**) Proliferation (viability) of HSF cultured in the medium pre-exposed to PCL-based film (MTS assay, 72 h, mean ± SD). (**b**) Proliferation (viability) of HSF cultured in peptide-modified cryogel (MTS assay, 72 h, mean ± SD). (**c**) Representative bright-field microscopy images of HSF 72 h post-seeding in the matrices.

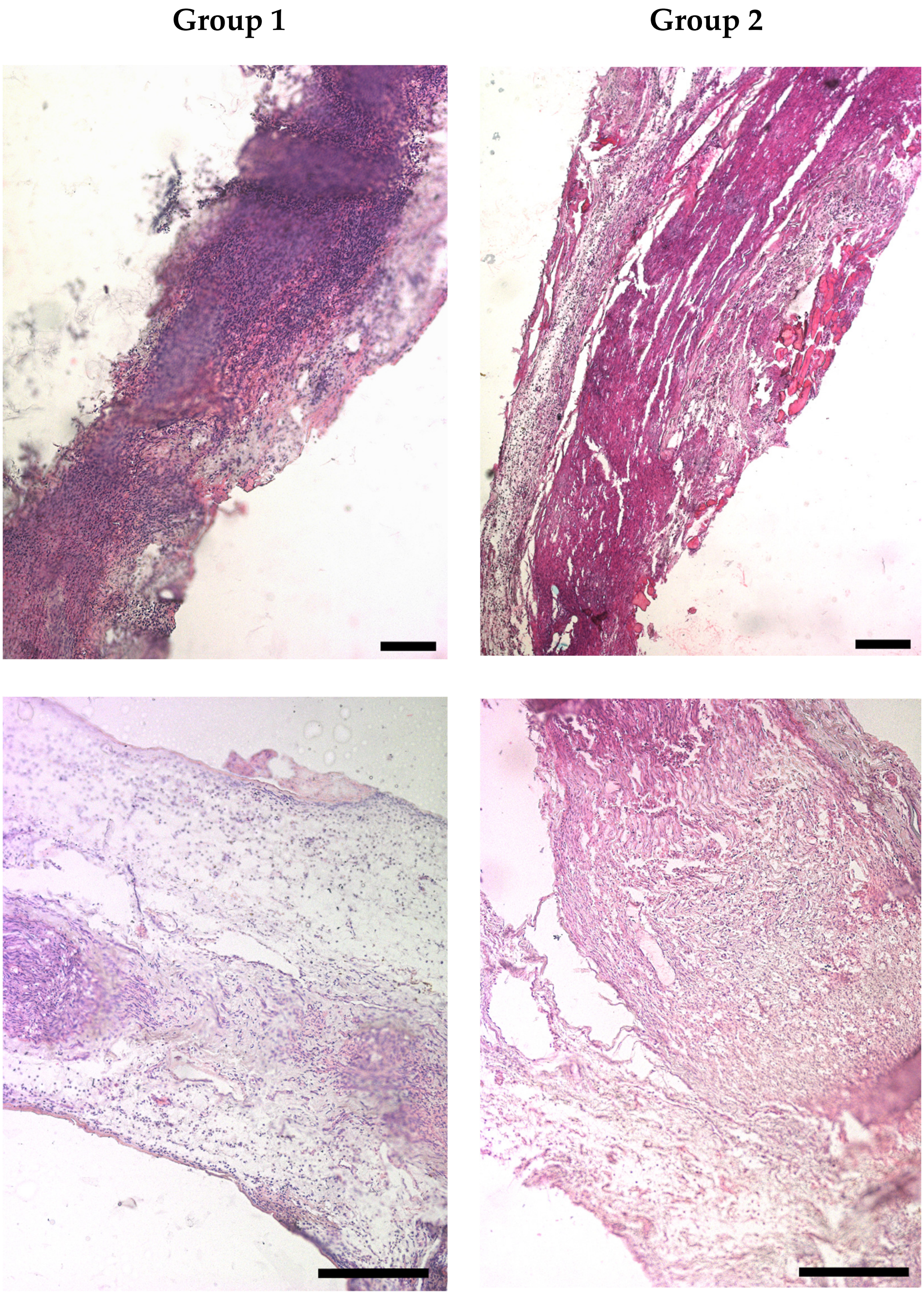

Figure S4. H&E-stained longitudinal sections of diastasis area (upper panel) and adjacent distal part of damaged sciatic nerve (lower panel) at different magnifications. Group 1 – hollow tube, group 2 – composite conduit (8 weeks). Scale bar is 500 μm, respectively.
